# Clinical Lectures upon Mental and Nervous Diseases

**Published:** 1869-12

**Authors:** 

**Affiliations:** Sainte Anne, Paris


A MONTHLY RECORD OF

*Medicine, Surgery, and the Collateral Sciences.*

EDITED BY J. ADAMS ALLEN, M.D., LL.D.; AND WALTER HAY, M.D.

VOL. XXVI. — DECEMBER, 1869. — No. 21.

### Original Translations.

ARTICLE I. — *Clinical Lectures upon Mental and Nervous Diseases; Alcoholism, Alcohol and Absinthe, Absinthic Epilepsy.* By M. MAGNAN, Sainte Anne, Paris.

(CONTINUED FROM PAGE 631).

§ III.—COMPARATIVE investigations with the different substances which enter into the composition of the absinthe-liquor, have been made under the most diverse conditions upon dogs, cats, rabbits, rats, guinea-pigs, and different birds. Since our first experiments, extending back to 1864, we have used, so far as it was possible, products of the same quality; we have procured from the house Chardin-Hadancourt, all the essences which we have used.

Physiological phenomena of little importance have resulted from using the essences of anise, star-anise, angelica, calamus aromaticus, origanum, fennel, mint and balm, although used in enormous doses, such as 15 to 20 grammes introduced into the stomach of a dog of medium size. The respiration was, generally, accelerated; the pulse became frequent; but the animal preserved his accustomed movements, he ate with good appetite and did not appear to be much inconvenienced.

During several hours, sometimes even during two or three days, the pulmonary exhalation diffused the peculiar odor of the substance ingested; the stools were likewise impregnated with the same odor. Essence of fennel placed under a bell-glass exhaled irritating vapors capable of producing lacrymation, cough, and sometimes salivation. In no case, let it be specially noted, did we detect *epileptic* or *epileptiform convulsions.*

It remains for us now to determine the effects produced by the essence of absinthe, and when we shall have been fully instructed in the action of each one of these substances taken separately, we shall see how certain phenomena are developed under the influence of mixtures of alcohol and essence of absinthe.

Whenever the essence of absinthe is absorbed, certain phenomena appear, whose duration and intensity are in direct ratio to the dose of the poison which has passed into the circulation.

Thus whilst a few centigrammes injected into the veins of a dog sufficed to develop symptoms, it was necessary to use three or four grammes, and sometimes more, when the poison was introduced into the stomach.

M. Magnan indicates the precautions to be taken in these experiments, the difficulties to be encountered according to the animals experimented with, according to the channel of absorption employed, whether the digestive tube (stomach or rectum), the pulmonary mucous surface, the cellular tissue or even the veins directly; whatever may be the route traversed by the poison in order to reach the nervous centres, the following symptoms will be observed:

When small doses of the essence of absinthe have been absorbed, there will be seen, soon afterwards, muscular tremors more or less marked, little, abrupt, interrupted shocks like electric discharges, repeating themselves one or more times in the muscles of the neck, and occasioning rapid and very circumscribed movements of the head, which is carried upward and backward; these contractions involve successively the muscles of the shoulders and of the back, and then induce abrupt shocks, exciting in spots and by jerks the anterior portion of the body; the animal cowers, gathers himself together, and seems to resist with all his force these powerful discharges. This is not all: under certain circumstances, in the case of the dog, a very interesting phenomenon supervenes.

The animal stops suddenly, remains motionless, as if stupefied, with lowered head, gloomy countenance, and drooping tail; and retains this attitude during one-half to two minutes, then resumes spontaneously his accustomed movements. This is a vertiginous state, which is not wanting in analogy to the "petit mal," or absent-mindedness of the epileptic.

The effect of the essence of absinthe in large doses is different, or rather there is a greater degree of intensity of the phenomena. After prodroma analogous to the symptoms just described, or even abruptly and more or less rapidly, according to the channel of introduction of the poison, attacks supervene in which the animal falls suddenly, with trismus, tonic convulsions predominating sometimes on one side of the body, and inducing a curvature, in which one side rests upon the ground by its middle portion, whilst the two extremities are elevated and bent upon the opposite side, approximate to each other.

To these tonic convulsions succeed, at the end of some minutes, clonic convulsions with chattering of the jaws, which interlock, or approximate convulsively without actually coming in contact entirely; next froth appears upon the lips, and sometimes the tongue is bitten; then stertorous respiration, evacuation of urine and fœcal matters, and sometimes of semen.

After the termination of the attack the animal retains, ordinarily, a slight degree of stupidity, but subsequently resumes his habitual condition; these attacks of epilepsy manifest themselves, sometimes, with this series of symptoms complete, having intervals between them of ten or twenty minutes or even more. Under the circumstances most frequently presented when the poison has been introduced into the stomach, a clear idea may be formed of the phenomena: but it is not always so; some symptoms may be wanting, or perhaps may succeed each other so rapidly as to be confounded. In some cases the attacks succeed each other without interruption, and thus there may be complicated, confused attacks with obstreperous convulsive rapid phenomena, in the midst of which the epileptic seizure is distinguished less clearly.

But as, in the same animal, after two or three isolated attacks, there may sometimes be seen to occur successive seizures which encroach upon each other in the manner of intercurrent accessions so as to constitute complicated attacks, by closely following the progress of the symptoms, the characteristics of the seizure may be detected in the midst of the confused scene. During the interval of the convulsive accessions, in the case of the dog, sometimes, real hallucinations may be witnessed. The animal elevates himself upon his paws, with bristling hair, frightened countenance, brilliant and blood-shot eyes staring fixedly, he barks furiously, advancing and receding as if in the presence of an enemy, then he becomes gradually more calm, and still growling for a few moments, finally becomes quiet. These phenomena, thus clearly outlined, only manifest themselves very rarely in the course of the experiments; but, however, the animal is frequently seen to exhibit, by his movements, a terror more or less acute.

In order to show you in a still more striking manner, the independence between the action of the cord and that of the brain, in the production of these two orders of facts, epileptic attacks on the one hand, fright and hallucination on the other, we shall subject to the action of absinthe, these two pigeons and this guinea-pig, from which the cerebral lobes have been removed. The convulsive attacks will be presented here, with the same characteristics as in those animals which have not undergone any mutilation. The poison acts, therefore, upon the entire cerebro-spinal system; but the appearance of cerebral phenomena in the intervals of the attacks, makes it evident that the action upon the different portions of the nervous centres can not be simultaneous; the brain, indeed, seems to enter into action when the other portions, being exhausted, are in repose, and reciprocally.

We have now acquired a tolerably complete notion of the comparative action of alcohol and of essence of absinthe; it remains to determine the effects produced upon the economy by the combination of these two agents administered at the same time.

§ IV.—If a mixture of alcohol and essence of absinthe be administered to an animal, at first alcoholic intoxication will be perceived, then the convulsive phenomena peculiar to absinthe, after a delay of one or more hours.

How is this tardy appearance of absinthic convulsions to be explained? We believe that it should be attributed to the action of the alcohol upon gastric digestion and absorption, an action which the experiments of M. Claude Bernard with ether and alcohol have perfectly demonstrated. Ether, you know, gentlemen, increases secretion, promotes digestion and absorption. Alcohol, on the contrary, diminishes and arrests secretion, retards digestion and absorption. It is no longer an excitant, it is an irritant.

I exhibit to you here, a dog, to which we have administered sixty grammes of alcohol, and four grammes of essence of absinthe. He is plunged, at this moment, into alcoholic intoxication. The absinthic convulsions will not be manifested until much later; nevertheless, the poison has already commenced to enter the circulation, since we can, by approaching the nostrils of this animal, determine, at each expiration, a decided odor of absinthe.

The delay in the gastric absorption, is not, however, the only cause which hinders the appearance of convulsive phenomena; there is, in addition, the action of the alcohol upon the nervous centres, rendering them temporarily less sensitive. The following experiment will furnish us a demonstration of this:

You have before you, a second dog, which, after having taken seventy grammes of alcohol, is perceived to be in a state of complete resolution. Disregarding the stomach, we will inject into a vein a few centigrammes of essence of absinthe. If the retardation of gastric absorption, due to alcohol, be the cause of the delay in the development of the symptoms. we shall see, in this case, the channel of absorption having been changed, the convulsive phenomena, at the end of a few minutes, as in those cases in which injection into the veins is performed upon a perfectly healthy dog, and, in fact, this is exactly what happens. You perceive the animal exhibits an epileptic attack; then, all is over, and we again recognize the condition of complete resolution. What does this experiment indicate? It shows us first, that, in the case of the first dog, the postponement of the convulsions was occasioned by the influence of the alcohol in the stomach. Next, that this postponement does not take place in a dog equally "alcoholized," when the absinthe is injected into a vein. In the second place, it demonstrates the influence of alcohol upon the cord, of which the excito-motor power seems diminished, since we have recognized in the alcoholized dog, but a single attack in place of a tolerably long continued succession of convulsive symptoms, as in the case of the animal submitted exclusively to the action of absinthe.

To sum up, the effects of alcohol and of absinthe are combined in the same subject, but a postponement and a slight diminution of intensity may be detected in the absinthic convulsions. In the case of animals dead in consequence of absinthic intoxication, the presence of the poison in the different organs may be determined by the odor. For this method of examination, certain precautions must be taken on account of the penetrating odor of the absinthe, which diffuses itself everywhere, and which would prevent the formation of very precise ideas of the impregnation of the organs by this substance, at least in the immediate locality of the autopsy. The cerebro-spinal membranes will be found to be injected, but it is rare to find sanguineous infiltrations in their thickness. It is generally in the middle of the medulla oblongata and in its vicinity, that the hyperæmia predominates. We have sometimes detected in the medullo-cervical region, a sanguineous infiltration of the pia-mater.

The brain cut into thin slices exhibits, like the cord, a faint diffused injection, without foci. The stomach presents, exceptionally, hæmorrhages into the thickness of its coats, whilst by the action of alcohol it becomes the seat, as we have seen, of lesions ordinarily very intense.

The pericardium, and in some cases the endocardium, presents little circumscribed ecchymoses, more numerous at the base of the heart, near the origin of the great vessels.

The lungs, usually slightly injected, may exceptionally, be infiltrated with blood at several points.

Nothing is found in other organs sufficiently constant, to deserve special notice.

The investigations which we have just made will enable us the better to appreciate in man the immediate effects of spirituous drinks, and to separate from simple acute alcoholism certain symptoms, such as the epileptic attacks, which never pertain to alcohol, but which depend almost exclusively upon absinthe. Whenever, accidentally, an individual drinks to excess, he becomes drunk. If these excesses are repeated several times, there is not much delay in the production of new phenomena, both intellectual and physical, and authors justly discriminate between drunkenness and acute alcoholic mania, or *delirium tremens*.

I shall recur, later, to the symptoms of drunkenness in man, as we shall compare the phenomena observed in animals, in order to demonstrate their identity. I shall not dwell, at this time, upon the particular condition designated by Percy by the title of *convulsive drunkenness*, in which the motor disturbances consist in clonic convulsions, very irregular, and without determinate character.*

Acute alcoholic mania, or *delirium tremens*, supervenes after repeated excesses in drinking, and ordinarily in persons habituated to drinking, when, by the aid of libations (potations?) more abundant than usual, they have exceeded their limit of saturation. It is upon this soil, thus prepared, that frequently acute symptoms are developed. It is the more necessary to bear this in mind, because you will perceive, in this fact, the explanation of certain phenomena which it would be difficult to attribute to intoxication. Sometimes, indeed, in the alcoholized, a multiple and variable delirium supervenes, but with which the special delirium of alcoholism manifests itself. This multiple delirium is found to be in relation to the cerebral state of the individual anterior to the last debauch. The alcohol may indeed conduce to the development of this multiple delirium, as an exciting cause, but never as an efficient cause. In other words, alcohol produces only alcoholic delirium; the other forms of delirium which may appear at times during alcoholism depend, I repeat it, not upon the poison itself, but upon the anterior state of the individual.

In those cases, again, in which the patient was primitively insane, the poison does not fail in its legitimate results; if the dose be sufficient, it invariably produces its peculiar delirium; this, however, is superadded to the anterior mental trouble, and by reason of its acuteness, in some cases, masks it completely, to the degree of rendering possible only the sole portion of the diagnosis referable to the alcoholic symptoms.

This is what happens, gentlemen, in general paralysis, especially during its first period; at this time, the symptoms of the disease are slightly marked, the physical disturbances are confused and disappear in the midst of the tumultuous trembling of the *delirium tremens;* the paralytic delirium, hardly sketched

* Ivresse Convulsive. *Dictionaire des Sciences Médicale*, 1818, p. 249.

out, is likewise obliterated before the prodigious activity of the alcoholic hallucinations. As the memory is wanting, it is necessary to await the diminution of these tumultuous phenomena, in order to perceive the different signs of general paralysis emerging.

You will have the opportunity to see, amongst the different patients which we shall examine together, two paralytics who have drunk to excess. In one of them we can not, at this moment, detect any thing beyond an attack of *delirium tremens*, if his perfectly well known antecedents did not aid us in completing the diagnosis; this, moreover, will become directly possible in a few days, when the alcoholic symptoms shall have amended. We will endeavor to determine the characters peculiar to acute alcoholic mania, or *delirium tremens.*

The symptoms arrange themselves naturally into two classes—intellectual disturbances, and physical disturbances. The phenomena on the part of the intellect consist especially in hallucinations, exceptionally of a joyous character, nearly always, on the contrary, as has been remarked long since, of a painful nature, and as M. Marcel says, determining moral impressions, of which the slightest would be astonishment, and the most vivid a profound horror.*

These hallucinations, according to their degree of intensity, according, likewise, to the disposition of the subject, give place to different reactions, capable of changing completely the physiognomy of the patient.

Hence, the maniacal, melancholic, stupid forms of alcoholic madness, forms which might be multiplied, but without real profit, if it were desirable to express all the aspects under which the disease can present itself. But whence does it happen that one and the same cause, a poison, determines symptoms so different?

These hallucinations, as we have said, which have for their common characteristic the awakening of painful sentiments, present, in their mode of expression, different degrees.

In the first degree, the patient thinks that he hears abuse and provocations; he sees thieves, policemen, animals, or even that

* *De la folie causée par l'abus des boissons alcoholiques.* Marcel. Thèse de 1847, p. 12.

he hears the voices of his parents, of his friends, who call him, who point out dangers, who invoke his assistance, etc.

Spurred on by these imaginings, the patient replies, threatens, quarrels, runs, dashes himself about, becomes furious, etc., quite as many acts which provoke in him turbulent manifestations, a maniacal condition.

Under other circumstances, he fancies himself in prison, before a tribunal; he is accused of different crimes, and sometimes imagines that he has committed them, believes himself deceived by his wife, by his friends, assists at the burial of his parents, etc. Under the weight of these sad impressions he is sombre, restless, defiant; he complains, is frightened, endeavors to fly, sometimes even conceives projects of homicide or of suicide; he appears, in a word, in the aspect of a melancholic.

Finally, at a point still more advanced, he imagines himself loaded with chains, at the foot of the scaffold; he sees before him the bloody corpses of children; all is on fire; he is about to be ingulphed, etc. These imaginations have crushed him, exhausted him; he remains motionless in a state of complete stupor.

Between these different conditions — maniac, melancholic, stupid — you understand, gentlemen, that numerous intermediate stages may be intercalated; but it will suffice for us to indicate it, by so much the more, that by following any one of the patients who are actually found in our wards, it will be possible to perceive, in the same subject, these different forms successively evolved. The direct examination of a patient will, much better than any description, impress upon the mind a clear idea of these varied transformations.

Permit me, gentlemen, to make a rapid analysis of these phenomena. But, first, it will not be uninteresting to dwell upon the development of those symptoms which do not present themselves from the beginning with such prominent characteristics.

A successive gradation, both in the intensity of the phenomena, and in their mode of evolution, may be observed. It proceeds from simple functional disturbance to illusion; from this to confused hallucination, which becomes by degrees distinct and precise hallucination, which is the highest degree. Then in proportion as amelioration progresses, the phenomena gradually disappear, following the same order; that is to say, precise hallucinations yield to confused hallucinations, these to illusions, which, in their turn, are followed by simple functional disturbance.

Such is the habitual evolution of hallucinations in the course of alcoholism. Exceptionally, however, they may reach their climax suddenly.

It is ordinarily at night that these accidents manifest themselves at first, and it is difficult to determine exactly their mode of evolution, in consequence of the confused recollection which patients preserve of them. I can at least indicate to you with certainty their mode of disappearance. Hallucinations at first persist during the day and night, commencing to disappear during the day, to be reproduced at night with the same degree of intensity; then becoming less clear, they appear at the moment of transition from wakefulness to sleep; next, only nightmare remains, which persists for a few moments after the subject awakens, then finally dreams; and, later still, the patient appreciates, with a certain degree of accuracy, all these sensorial troubles, which terminate by disappearing completely. Thus, at first, hallucinations by day and night, then only at night; later, confused hallucinations and illusions at the moment of transition from wakefulness to sleep; then during sleep, with restless awakening; then, lastly, nightmares, dreams, and restoration to health.

It will be easy for you, gentlemen, to follow this retrograde movement in the majority of patients, however imperfectly you may fix your attention upon this point. In proportion as they are cured, they remark to themselves, with pleasure, the favorable changes which occur, and some are capable of recalling them in a very graphic manner.

Let us see, now, how these phenomena are developed, in each direction. To the hearing, the first sensations are roaring, whistling, tinkling, ringing of bells, which the patient imagines a funeral toll; next, confused noises, crashing, tumults, riots, fusillades; still later, cries of distress, confused voices, distinct voices, those of friends, of parents; then, again, abuse, menaces, accusations, clearly stated, etc., aural hallucinations the most distinct.

To the sight, the symptoms present themselves in the same manner; vision is disturbed, is obscured with mists, it perceives sparks, flames, various colors, shadows, tremulous objects, phantoms, then confused sensations of conflagrations, of precipices, of battles. In some cases, the patient perceives a dark spot, blackish, with diffused margin, then with distinct limits and prolongations, which become paws, and a head, and form an animal, a rat, a cat, a lion, etc., soldiers, police, assassins, etc. These are a few of the complicated troubles which are imposed upon the vision of the drunkard.

The perversions, illusions and hallucinations of the senses of taste and smell, are less numerous and less varied; nevertheless the drunkard escapes neither offensive tastes nor odors.

The general sensibility, with its different phases of anæsthesia and hyperæsthesia, furnishes, likewise, its contingent of painful sensations, and frequently its disturbances are associated with those of the other senses. Thus some inebriates see and feel animals penetrating and tearing their skin and flesh; or themselves surrounded by bands of iron which inlace, inclose and oppress them. They busy themselves in untwisting these metallic meshes, which incessantly reunite; or they may even see a portion of their bodies gnawed by worms. They pick them off, and struggle, in the midst of the greatest anxiety, to detach and cast them to the earth. I shall not attempt to picture to you the anguish of these wretches; every inebriate who is presented to you will enable you to complete the somewhat hurried enumeration which I have made of these symptoms.

I shall not dwell, to-day, upon certain disturbances, which involve digestion and nutrition. I will, however, say, concerning the condition of the urine, that according to our physiological experiments, and especially according to our clinical investigations, the presence of albumen in the urine must be considered a very unusual incident in acute alcoholism.

The phenomena which occur in connection with mobility, are very remarkable; the shivering of the whole body, the general tremor, the convulsive trembling of all the muscles of the face, of the hands, of the legs, are exhibited to this degree in acute alcoholism alone. It is not necessary, in order to observe them, to exact from the patient certain movements, certain positions demanding a little more certainty, and more favorable, consequently, to the appreciation of the disorder. The most simple movements suffice to render evident the intensity of the motor trouble. If the patient seizes you, you perceive that his fingers press in an irregular manner, so that the hand closes convulsively, or else a sort of arrest occurs.

Should he attempt to speak, in the midst of the muscular quivering, slight convulsive jerks may be observed, giving the countenance an expression of grimace. When the arms are extended, besides the trembling in mass, little, irregular, unequal shocks manifest themselves in the greater portion of the muscles. In some parts the tendons become abruptly prominent, and by the application of the hand very irregular contractions will soon be detected.

In the opinion of most physicians, these very considerable lesions of mobility are the necessary preliminary steps toward convulsive epileptic symptoms. After these, consequently, the epileptic crisis should be developed as the last term of the muscular disorders of acute alcoholism, and would always be the index of a grave condition. It is not so, gentlemen; the epileptic attack is not, in acute alcoholic mania, the highest expression of motor disturbance; it is an accident independent of the other phenomena Certain patients indeed, sometimes, exhibit tremor of frightful intensity, and, nevertheless, they manifest no epilepsy. Others, on the contrary, have almost no tremor, and you perceive them suddenly surprised by the attack. These occurrences will no longer astonish you; you understand their explanation.

